# A Novel RUNX1 Genetic Variant Identified in a Young Male with Severe Osteoporosis

**DOI:** 10.1002/jbm4.10791

**Published:** 2023-07-29

**Authors:** Tomasz J. Block, Cat Shore‐Lorenti, Roger Zebaze, Peter G. Kerr, Anna Kalff, Andrew Charles Perkins, Peter R. Ebeling, Frances Milat

**Affiliations:** ^1^ Department of Endocrinology Monash Health Melbourne Victoria Australia; ^2^ Department of Diabetes, Central Clinical School Monash University Melbourne Victoria Australia; ^3^ Centre for Endocrinology and Metabolism Hudson Institute of Medical Research Clayton Victoria Australia; ^4^ Department of Medicine, School of Clinical Sciences Monash University Melbourne Victoria Australia; ^5^ Department of Nephrology Monash Health Melbourne Victoria Australia; ^6^ Department of Haematology Alfred Health Melbourne Victoria Australia

**Keywords:** ANABOLIC THERAPY, IDIOPATHIC OSTEOPOROSIS, OSTEOGENESIS, OSTEOPOROSIS IN YOUNG ADULTS, RUNX1 GENETIC VARIANT

## Abstract

This case describes a young man with an unusual cause of severe osteoporosis and markedly deranged bone microarchitecture resulting in multiple fractures. A potentially pathogenic germline variant in the runt‐related transcription factor 1 (RUNX1) gene was discovered by a focused 51‐gene myeloid malignancy panel during investigation for his unexplained normochromic normocytic anemia. Further bone‐specific genetic testing and a pedigree analysis were declined by the patient. Recent experimental evidence demonstrates that RUNX1 plays a key role in the regulation of osteogenesis and bone homeostasis during skeletal development, mediated by the bone morphogenic protein and Wnt signaling pathways. Therefore, rarer causes of osteoporosis, including those affecting bone formation, should be considered in young patients with multiple unexpected minimal trauma fractures. © 2023 The Authors. *JBMR Plus* published by Wiley Periodicals LLC on behalf of American Society for Bone and Mineral Research.

## Graphical abstract

A young man with a normochromic normocytic anaemia, severe osteoporosis and deranged bone microarchitecture resulting in multiple fractures associated with a potentially pathogenic germline variant in the runt‐related transcription factor 1 (RUNX1) gene. As RUNX1 plays a key role in skeletal development, further clinical investigation in this area is warranted.

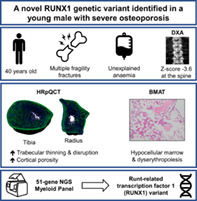

## Introduction

Osteoporosis in younger adults (premenopausal women and men aged less than 50 years) is often challenging to diagnose and treat due to several factors, including a poor understanding of the underlying pathophysiology, lack of unified guidelines for diagnosis and management, and limited research in this area.^(^ ^1^ ^)^ Although many such cases are due to underlying primary or secondary causes, some patients present without an identifiable cause, termed idiopathic osteoporosis (IOP), which has been associated with abnormal bone microarchitecture.^(^ ^2^ ^)^ It is thought that this patient cohort may have a yet uncharacterized primary genetic basis, with previous molecular screening of young osteoporotic adults revealing several rare known or novel pathogenic gene variants.^(^ ^3^, ^4^ ^)^ However, despite these findings, many patients undergoing genetic screening analysis do not have identifiable mutations in causal genes, suggesting that further research into this area is required. Here we describe osteoporosis with impaired bone microarchitecture in a young man with a novel and potentially pathogenic germline genetic variant in runt‐related transcription factor 1 (RUNX1).

## Clinical Vignette

A 40‐year‐old male presented for review of osteoporosis in the setting of several fractures, including clavicle (high‐impact), at age 16 years, as well as minimal trauma ankle and wrist fractures in his early twenties. Following a vertebral and elbow fracture sustained from a low‐velocity fall at age 39 years, severe osteoporosis was diagnosed on dual energy X‐ray absorptiometry (DXA) with a bone mineral density (BMD) Z‐score of −3.6 at the spine (0.685 g/cm^2^) and −2.3 at the hip (0.659 g/cm^2^). A family history for osteoporosis was positive in his mother who had had a minimal trauma ankle fracture in her fifties. He had no glucocorticoid exposure or other chronic illness. A spine MRI at age 39 showed T6 and T7 vertebral compression fractures, without any evidence of skeletal dysplasia on imaging of the spine, pelvis, or hips. He had also undergone mandibular surgery for idiopathic condylysis in the past and had normal facies without body disproportion and no other dental abnormalities, hyper‐ or hypomobility, skin lesions, deafness, or ophthalmological issues. He was a nonsmoker with minimal alcohol intake, with a weight of 58 kg, height 168 cm, and a BMI of 20.5 kg/m^2^.

An extensive screen for secondary causes of osteoporosis was negative, apart from mild vitamin D deficiency (40 nmol/L), which was promptly corrected. Investigations revealed a normal serum tryptase and full blood count. The serum amino‐terminal propeptide of type 1 collagen (P1NP) bone formation marker was 56 μg/L (N 15–80 μg/L)^(^ ^5^ ^)^ and serum β cross‐linked C telopeptide (CTX) was 388 ng/L (N 100–600 ng/L); the serum 25‐hydroxyvitamin D had increased to 88 nmol/L. Serum‐corrected calcium was 2.26 mmol/L (N 2.15–2.55 mmol/L), phosphate was 1.05 mmol/L (N 0.8–1.5 mmol/L), parathyroid hormone (PTH) was 5.2 pmol/L (N 1.6–6.9 pmol/L), alkaline phosphatase was 50 U/L (N 25–110 U/L), and other liver function tests, as well as thyroid function tests and glucose and kidney function (eGFR >90 ml/min/1.73m^2^), were all normal, and his testosterone was normal, as was the erythrocyte sedimentation rate (ESR). Serum coeliac disease autoantibodies (anti‐gliadin IgG, t‐transglutaminase IgA) were negative, as were serum and urine electrophoresis. Spot urinary calcium and creatinine were 3.1 and 2.9 mmol/L respectively, with a raised calcium/creatinine ratio of 1.07 mmol/mmol (N 0.1–0.5 mmol/mmol). In addition, a radionuclide bone scan (technetium Tc 99 m hydroxydiphosphonate) did not demonstrate any abnormal uptake.

Treatment comprised optimizing dietary calcium and initiating regular progressive resistance training exercise and commencing weekly 35 mg oral risedronate. His BMD improved, with a spinal Z‐score of −2.8 (+ 0.064 g/cm^2^) and −1.9 (+ 0.065 g/cm^2^) at the hip within 2 years. At age 43, he had increased lethargy and malaise with associated weight loss of 8 kg over several months, with the diagnosis of a new normochromic and normocytic anemia with a Hb of 95 g/L (baseline 130 g/L). General examination was unremarkable; he was normotensive with a standing blood pressure of 114/75 mmHg, and there were no rashes or arthropathies. Further investigations revealed microscopic hematuria with glomerular red blood cells in the urine and an elevated serum creatinine of 117 μmol/L (baseline 78 μmol/L) and mild proteinuria of 280 mg/d (N < 165 mg/d) with an elevated albumin/creatinine ratio (ACR) of 14.0 mg/mmol (N < 2.5 mg/mmol). Anti–neutrophil cytoplasmic antibodies (ANCA), a further serum myeloma screen, and anti–nuclear antibodies (ANA), anti–double–stranded deoxyribonucleic acid (dsDNA), rheumatoid factor (RF), and hepatitis serology were negative, and an abdominal ultrasound was normal.

**Fig. 1:**
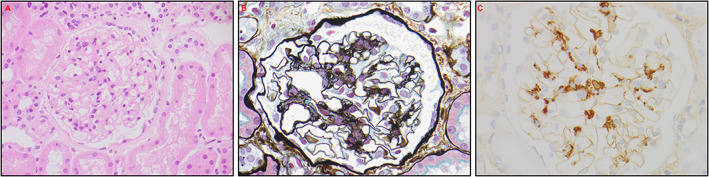
Renal biopsy. (*A*): Hematoxylin and eosin stain demonstrating mild mesangial matrix expansion without significant capillary hypercellularity. (*B*): Silver stain again demonstrating mesangial matrix expansion. (*C*): IgA staining with mild positivity.

**Fig. 2:**
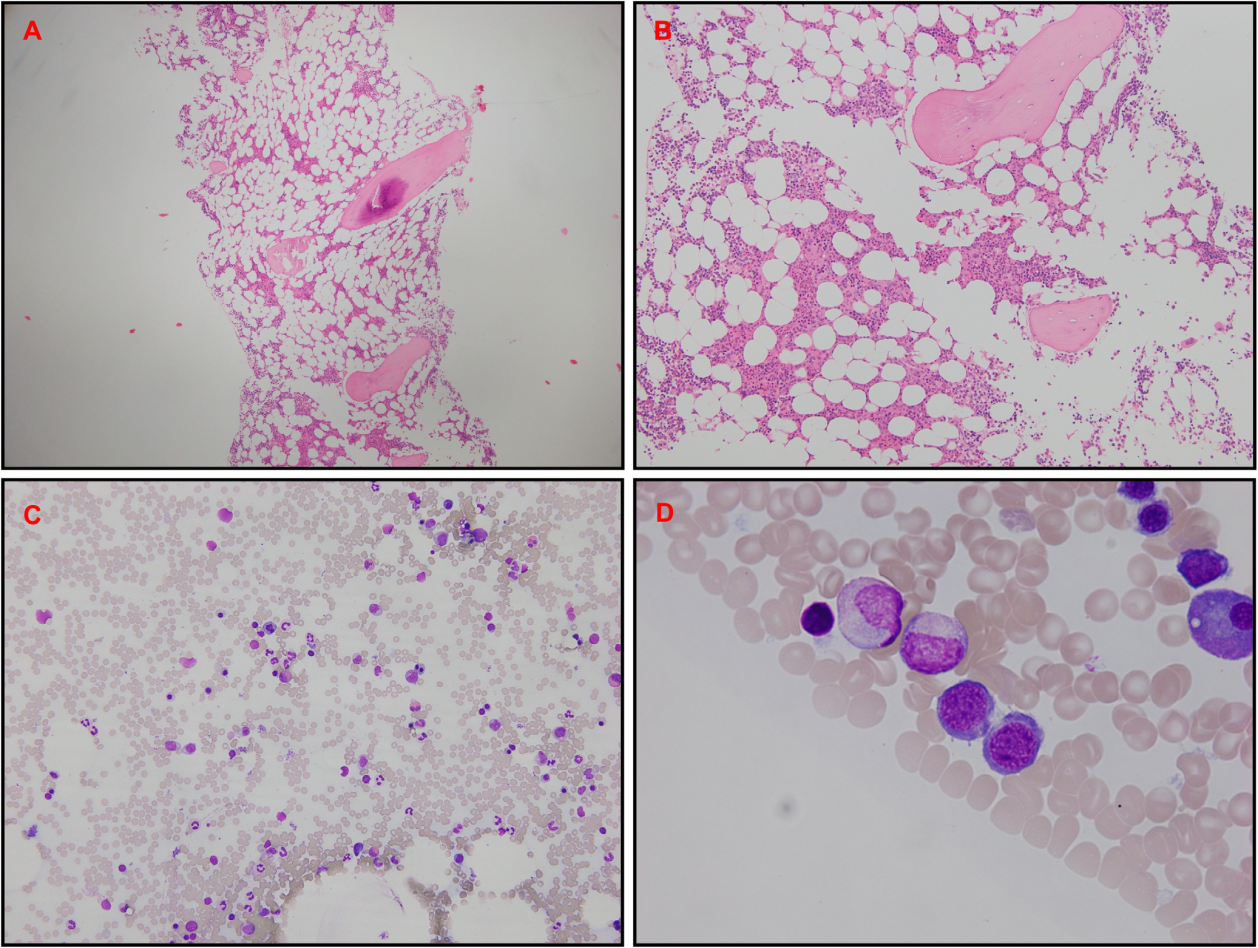
Bone marrow biopsy. (*A* and *B*) Hematoxylin and eosin trephine stain demonstrating mild to moderate hypocellular marrow (20%–30%) with moderately reduced erythropoiesis. (*C* and *D*) Aspirate demonstrating mild dyserythropoiesis with normal granulocytic and megakaryocytic lineages.

**Fig. 3:**
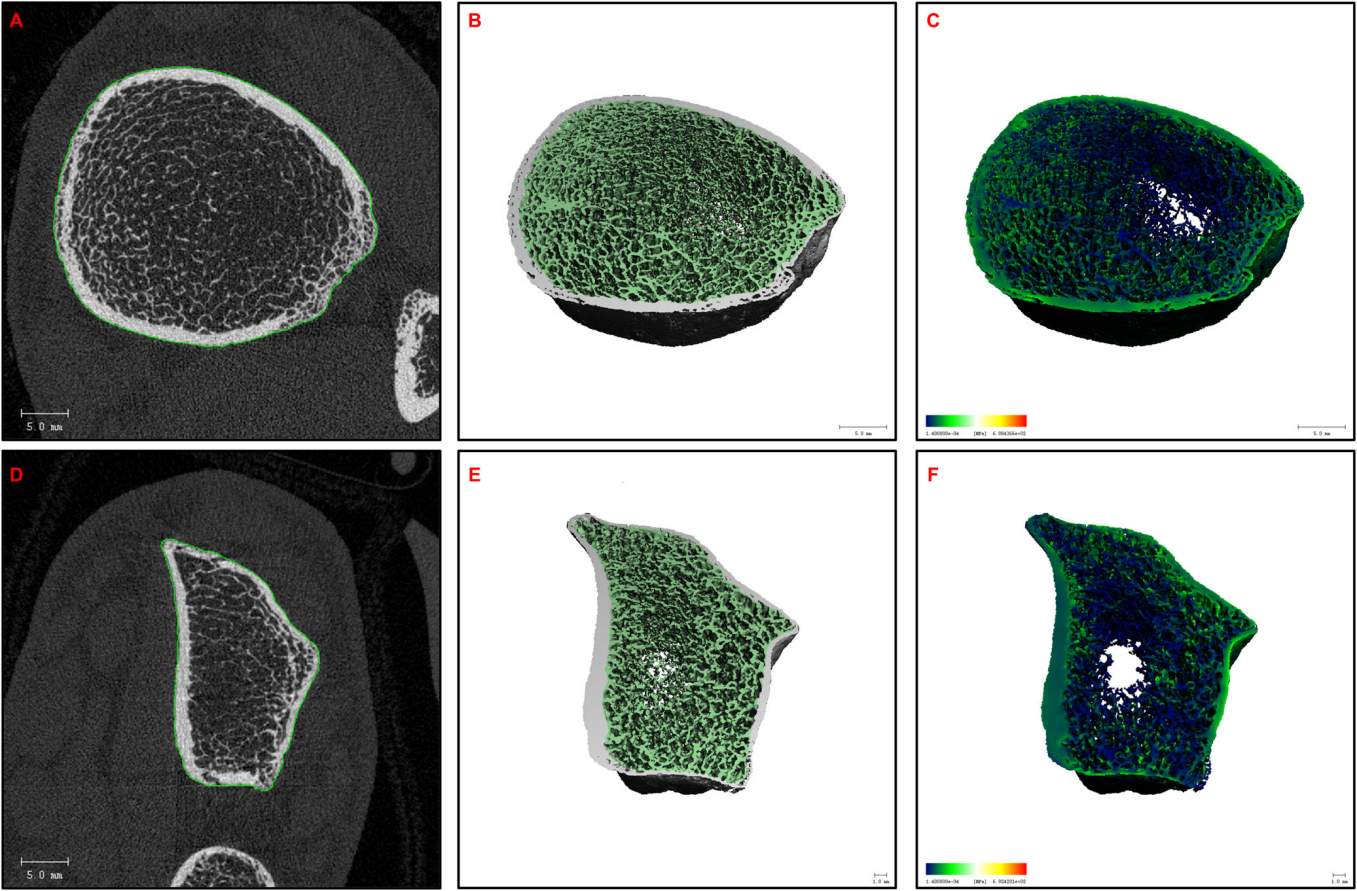
High‐resolution peripheral quantitative computed tomography. (*A*–*C*) Left tibia and (*D*–*F*) left radius, both demonstrating trabecular thinning and disruption, with increased cortical porosity.

**Fig. 4:**
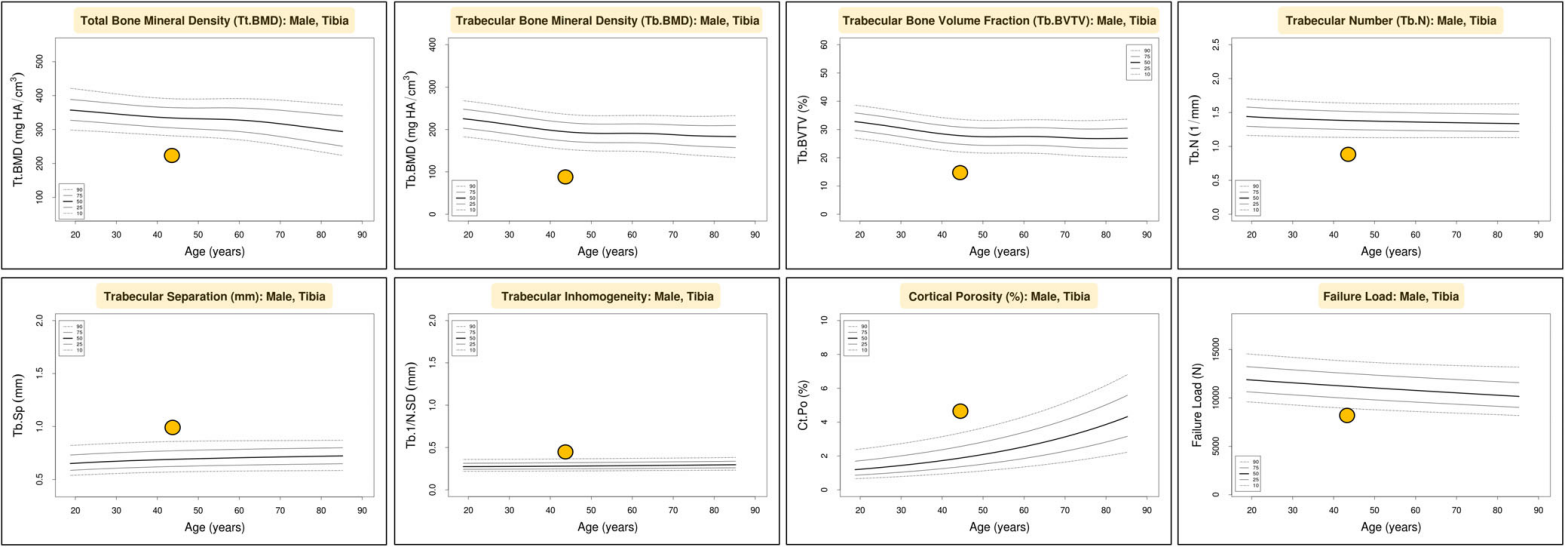
Left tibia HR‐pQCT normative data and percentile graphs (analyzed at www.normative.ca).

**Fig. 5:**
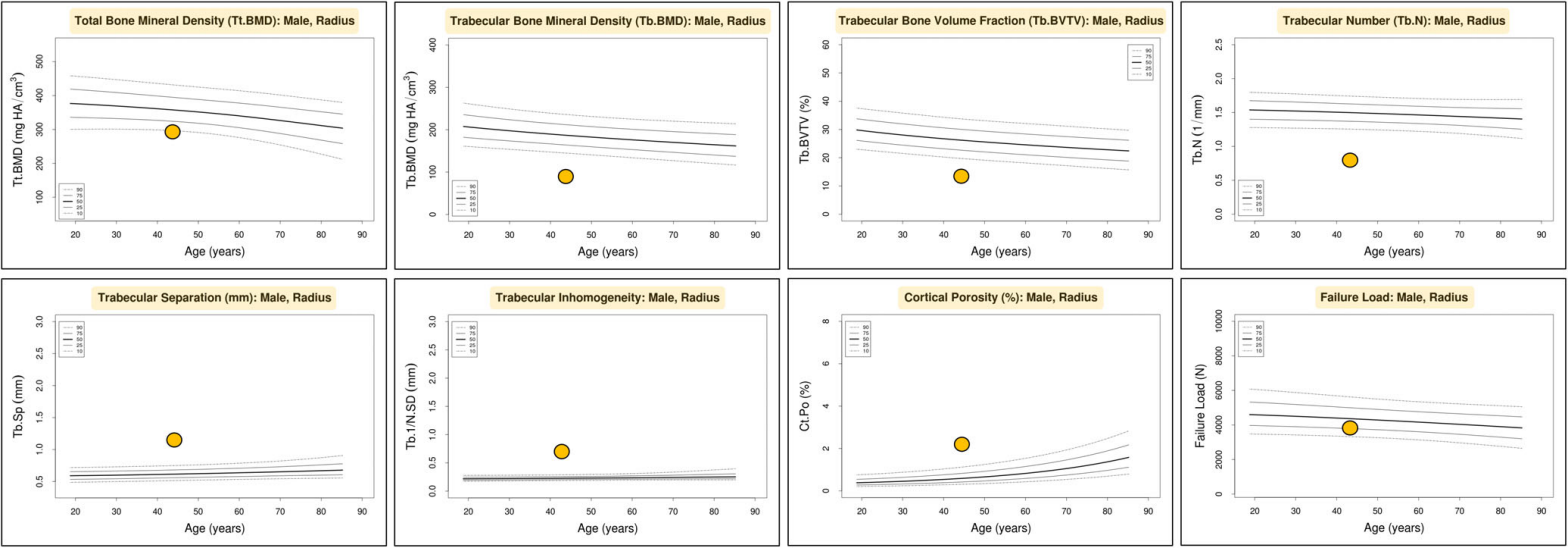
Left radius HR‐pQCT normative data and percentile graphs (analyzed at www.normative.ca).

**Fig. 6:**
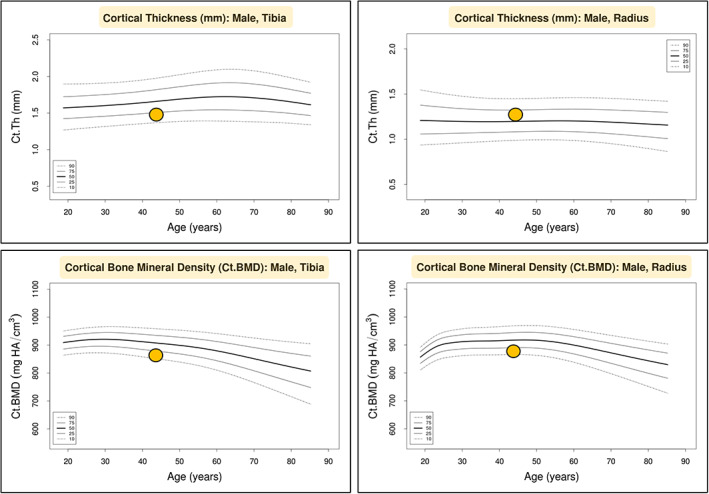
Left tibia and left radius cortical bone thickness and cortical density HR‐pQCT normative data and percentile graphs (analyzed at www.normative.ca).

A subsequent renal biopsy demonstrated mild IgA nephropathy (IgAN) (Fig. 1), and he was commenced on candesartan 16 mg daily for long‐term renal protection. However, given his ongoing unexplained mild normochromic normocytic anemia, a hematology review in the following year recommended a bone marrow aspiration and trephine biopsy (BMAT), which demonstrated a hypocellular marrow and dyserythropoiesis. (Fig. 2). There was no evidence of acute myeloid leukemia (blasts <2%), and the myeloid‐to‐erythroid ratio was 1.9. In addition, a 51‐gene next‐generation sequencing (NGS) myeloid malignancy panel (Methods) identified a heterozygous germline RUNX1 single nucleotide variant (Arg250His). In view of a likely beneficial effect on osteoblast dysfunction, the patient was transitioned to the anabolic agent teriparatide 20 μg daily. A high‐resolution peripheral quantitative computed tomography (HRpQCT) scan of the left tibia and left radius prior to teriparatide commencement demonstrated profound reductions in trabecular volumetric bone density (Tb.vBMD), trabecular bone volume fraction (Tb.BVTV), and trabecular number (Tb.N) (all lowest second centile for age), as well as increased trabecular separation (Tb.Sp), trabecular inhomogeneity (Tb.1/N/SD), and intracortical porosity (Ct.Po) (all highest second centile for age). In addition, tibial and radial cortical bone thickness (Ct.Th) were in the lowest 20th and 50th centiles for age, respectively, while tibial and radial cortical bone densities (Ct.BMD) were both in the lowest 20th centiles for age, and tibial and radial failure load (N) was in the lowest 10th and 30th centiles for age, respectively (Figures 3, 4, 5, 6).

The patient will have close clinical follow‐up with hematology as he continues to have an isolated normochromic normocytic anemia, as well as ongoing renal and endocrinology review. His response to teriparatide will be monitored with repeat DXA and HRpQCT at 12 months.

## Discussion

RUNX1 plays a critical role during organogenesis, including definitive hematopoiesis^(^ ^6^ ^)^ and skeletal development.^(^ ^7^, ^8^ ^)^ A wide range of both germline and somatic RUNX1 genetic variants have been associated with various myeloid and lymphoblastic malignancies.^(^ ^9^ ^)^ Genetic screening has revealed that RUNX1 is one of the most frequently altered genes in myelodysplastic syndromes (MDS)^(^ ^10^ ^)^ and, when present in acute myeloid or lymphoblastic leukemia, is associated with an overall poorer prognosis.^(^ ^11^, ^12^ ^)^

From a bone viewpoint, RUNX1 induces skeletal progenitor cells into early stages of chondrogenesis,^(^ ^13^ ^)^ is involved in sternal development,^(^ ^14^, ^15^ ^)^ regulates endochondral ossification during fracture healing,^(^ ^16^ ^)^ and upregulates several bone‐specific genes associated with bone formation and homeostasis in trabecular and cortical bone.^(^ ^17^ ^)^ However, despite the detection of RUNX1 in osteoblast progenitors, preosteoblasts, and mature osteoblasts, the exact mechanisms regulating bone maturation are unknown, and our understanding of the role of RUNX1 in bone formation is evolving.

Recent experimental evidence utilizing RUNX1 conditional knockout mice has revealed that RUNX1 enhances osteoblast differentiation, thereby promoting bone formation and inhibiting adipogenesis by upregulating the Bmp7/Alk3/Smad1/5/8/Runx2/ATF4 and WNT/β‐catenin signaling pathways, which are involved in bone formation to maintain postnatal and adult bone homeostasis.^(^ ^18^ ^)^ Specifically, it was shown that RUNX1 deficiency impaired both BMP and TGF‐β signaling in the femur, tibia, and calvarial cells of the knockout mice, in addition to impaired β‐catenin signaling with a downregulation of active β‐catenin protein levels in trabecular bone. This led to reduced bone density, bone volume, and trabecular number and an increase in trabecular bone separation, which parallel the HRpQCT findings demonstrated in our patient. In RUNX1 conditional knockout mice, serum P1NP was decreased but CTX levels were unchanged compared with wildtype mice. Interestingly, in an ovariectomized animal model used to simulate estrogen depletion‐induced osteoporosis, RUNX1 overexpression via an adeno‐associated virus (AAV)‐mediated gene expression significantly increased bone volume.

Given the emerging role of RUNX1 as a central regulator of osteogenesis, we suggest that the RUNX1 variant identified in this patient with a normochromic normocytic anemia and severe osteoporosis is potentially pathogenic. To our knowledge, this is the first case study to report human skeletal bone abnormalities in the context of a heterozygous germline RUNX1 variant. This single‐nucleotide missense variant is c.749G > A with respect to NCBI Reference Sequence NM_001754.5, replacing arginine with histidine, both of which are basic and polar, at codon 250 of the RUNX1 protein (p.Arg250His). The RUNX1 protein is highly conserved between mouse, rat, and human species, with 100% conservation for the region that encompasses the Arg‐250 amino acid.^(^ ^19^ ^)^ The variant is in a disordered domain C‐terminal to the DNA‐binding domain with a cytogenic location of 21q22.12 and has no well‐described function. It is unclear whether this region is established as a transactivation domain (TAD); it is likely between the DNA‐binding domain and the TAD.^(^ ^9^ ^)^ This variant (rs771614642) is rare, with an allele frequency of 0.000046 in a large population dataset (gnomAD version 3.1.2: 7/151250 total alleles; no homozygotes). Ensemble missense variant prediction analysis suggests a potential deleterious effect on protein structure/function (REVEL score: 0.63; CADD score: 25.7).^(^ ^20^, ^21^, ^22^ ^)^ It is currently classified as a variant of uncertain significance (VUS) with multiple listings in the ClinVar human genetic variant database (Variation ID 239055)^(^ ^23^ ^)^ and one listing associated with MDS^(^ ^24^ ^)^ in the catalogue of somatic mutations in cancer (COSMIC, mutation COSV55881639).^(^ ^25^, ^26^ ^)^ Further genotypic and phenotypic characterization of the patient's variant is unavailable as extended segregation analysis has been declined in the patient's extended family, including in his young children. However, the patient's RUNX1 variant was also observed in a recent clinical study sequencing DNA of 4836 children with newly diagnosed B‐cell acute lymphoblastic leukemia (B‐ALL), with two children harboring the germline RUNX1 Arg250His missense variant.^(^ ^27^ ^)^ In particular, this variant demonstrated a 19% loss in transcription activator activity compared with the wildtype protein using the luciferase reporter assay in HeLa cells, with similar protein levels as compared to wildtype RUNX1 in HeLa cells, and was therefore not deemed to have a significant effect on RUNX1 gene function, so it was not studied further. It is unknown whether the two children with the variant in this study had any phenotypic characteristics suggesting skeletal bone abnormalities. In this patient, the NGS panel was designed to investigate suspected myeloid malignancies^(^ ^28^ ^)^ and so did not include all known pathogenic variants related to osteopenia. RUNX1 was assigned as the causative variant as no other variant of significance was found. Further functional studies, such as a DNA‐binding assay or reporter assay, would be helpful in assigning causality more strongly to this RUNX1 variant.

Recent genetic sequencing studies of patients with IOP identified a small subset of patients with either known or likely pathogenic gene variants or variants of undetermined significance, affecting defects in bone formation or resulting in high bone turnover.^(^ ^3^, ^4^, ^29^ ^)^ A previous meta‐analysis of genome‐wide association studies (GWAS) in human individuals revealed numerous gene loci linked with BMD variation and risk of fracture.^(^ ^30^ ^)^ Among those found to be most significant was runt‐related transcription factor 2 (RUNX2), which is a known osteoblast‐specific transcription factor involved in skeletal bone formation and osteoblast differentiation.^(^ ^8^, ^31^ ^)^ Indeed, population studies have shown that RUNX2 genetic variation is associated with differences in BMD and risk of fracture.^(^ ^32^, ^33^ ^)^ However, no such evidence exists for RUNX1 polymorphisms exerting human low BMD phenotypes. Given the often ubiquitous and transcriptional nature of the affected genes, extraskeletal manifestations affecting other organ systems have been described.^(^ ^4^ ^)^ In our patient, it is possible that his hematological and nephrological conditions are related to the known RUNX1 variant. Interestingly, a novel Mendelian randomization study provided evidence that the hematopoietic system may regulate the skeletal system in humans, with red and white blood cell traits observed to have positive and inverse causal effects on BMD, respectively.^(^ ^34^ ^)^ Given the known association of RUNX1 genetic variants in the pathophysiology of human MDS, it is possible that this patient's bone marrow and aspirate findings represent an early “phenotype” of lower‐risk MDS, which may progress in the future due to disruption of antitumor cellular defense.^(^ ^35^ ^)^ While the patient does not currently meet the criteria for RUNX1 familial platelet disorder with associated myeloid malignancies (FPDMM) or acute myeloid leukemia (AML), the reported pathogenic variants for these disorders are unlike the variant in this case.^(^ ^36^ ^)^ Although the exact etiology of primary IgAN in humans remains unknown, this polygenic disease is thought to have a significant autoimmune basis with dysregulated IgA synthesis involving both CD4+ (T‐helper) T cells and B lymphocytes (CD5+ CD19+ B cells).^(^ ^37^ ^)^ Notably, RUNX1 is involved in multiple crucial roles in various immune cell subsets, including the determination of T cell (CD4+/CD8+) lineage choice and early B‐cell development.^(^ ^38^ ^)^ Whether this young male's IgAN is related to the detected RUNX1 variant is unclear. Evidently, in this patient, there are marked alterations in bone microarchitecture, indicating a possible defect in cortical and trabecular bone formation, as well as bone quality, despite bone turnover markers being in the normal range. Thus, the precise mechanisms underlying these changes await elucidation.

The hypothesis that disorganized bone formation contributed to the pathogenesis of bone fragility is supported by the presence of marked cortical porosity in this relatively young patient. Classically, increased porosity is due to a bone‐remodeling imbalance leading to bone loss and structural decay in the cortical compartment with advancing age.^(^ ^39^ ^)^ Metaphyseal cortical bone is formed via corticalization of trabeculae adjacent to the cortex during growth, with disruption of this process resulting in abnormal cortical bone with increased cortical porosity.^(^ ^40^ ^)^ As a result of the disorganization of bone components with poor interconnection, load is ineffectively transferred, causing tissue damage and subsequent inflammation triggering a cascade of biological and anatomopathological events leading to bone abnormalities, fragility, and ultimately, fractures.^(^ ^41^ ^)^ This patient's previous history of condylar resorption may represent yet another clinical manifestation of ineffective load conduction in a site of intense, repetitive, and unpredictable mechanical loading.^(^ ^42^ ^)^ Such fractures may occur independently of bone density and architecture.

Therefore, in a young patient with multiple minimal trauma fractures, it is imperative to consider rarer causes of osteoporosis, especially when they appear in a constellation with other clinical signs and symptoms. Successful identification of an underlying cause can lead to appropriate preventive care, which may also include surveillance and/or treatment of associated and clinically significant conditions. Impaired bone formation should also be considered, and further investigations to assist a diagnosis may include HRpQCT, NGS, assessment of the quality or extent of disorganization of bone, and bone biopsy. In the context of a condition where the primary problem is osteoblast dysfunction, together with profound reductions in BMD, anabolic agent therapy could also be considered. By analogy with the preclinical studies of RUNX1 overexpression, there may also be a role for RUNX1 as a potential therapeutic target for novel osteoporosis therapy.

## Methods

All serum chemistries were performed at Melbourne Pathology, NATA Accreditation No. 2133, Site No. 2126.

Dual energy X‐ray absorptiometry measurements were performed on the same machine at both time points (Hologic, Horizon) at I‐MED Radiology Network, Caulfield Hospital, VIC 3162, Australia.

Renal biopsy histological analysis performed at Monash Health Pathology, NATA Accreditation No. 2762. Site No. 2755.

High‐resolution peripheral quantitative computed tomography images acquired at the distal radius and tibia using Xtreme CTII (Scanco Medical AG, Switzerland, voxel size 60 μm) as per standard protocol. The scan region of interest is 9.5 mm proximal to the radial midjoint and 22.5 mm proximal to the tibial midjoint.

Bone marrow aspiration and trephine biopsy processed at Alfred Health Pathology, NATA Accreditation No. 2349, Site No. 2342. Reported by Hematologist Dr Anna Kalff, who has a special interest in malignant hematology.

Next‐generation sequencing custom myeloid panel conducted on genomic DNA and performed at Alfred Health Pathology, NATA Accreditation No. 2349, Site No. 2342. Curation is per ACMG guidelines and reporting by molecular pathologists. The panel has 51 genes that include all of those recommend by an expert curation team from the Australian Leukaemia and Lymphoma Group (ALLG).

Exact DNA was extracted from the bone marrow. Libraries were prepared from genomic DNA according to the SureSelect XT HS2 DNA System with Pre‐Capture Pooling manual (Agilent). Quality and quantity of libraries were determined by TapeStation using D1000 ScreenTape (Agilent). Sequencing was performed on an Illumina Miniseq platform (Illumina) on Mid output 300‐cycle cartridge (2 × 150bp).

Gene panel targets: All exons in the following genes are covered unless otherwise specified: ASXL1, BCOR, BCORL1, CALR (NM_004343; exon 9), CBL (NM_005188; exon 2–3, 6–10, 13, 15), CEBPA, CISH, CSF3R (NM_156039; exon 14, 17), DNMT3A, EPOR, ETV6, EZH2, FLT3 (NM_004119; exon 14–17, 20–21), FOXO3, GATA2, GNAS (NM_000516.5, exon 2–13), IDH1 (NM_005896; exon 4, 7–9), IDH2 (NM_002168; exon 4, 7), JAK1 (NM_002227; exon 14), JAK2 (NM_004972; exon 1–14, 16–25), JAK3 (NM_000215.3; exon 1–12, 14–22), KIT (NM_000222; exon 8, 11, 13, 14, 17, 18), KLF3, KRAS (NM_033360; exon 2–4), MPL, NF1, NFE2, NPM1 (NM_002520; exon 11), NRAS (NM_002524; exon 2–3), PHF6, PPM1D, PTPN11 (NM_001330437.2; exon 3, 8, 11–13), RAD21, RUNX1, SETBP1 (NM_015559; partial exon 4), SF3B1 (NM_012433; 12, 14–15), SH2B3, SMC1A, SMC3, SOCS1, SOCS2, SOCS3, SRSF2 (NM_003016; exon 1, 2), STAG1 (NM_005862.3; exon 1, 3–8, 10–33), STAG2, STAT5B (NM_012448.3; exon 1–6, 8–18), TET2, TP53, U2AF1 (NM_006758; exon 2, 6), WT1 (NM_024426; exon 6–8), ZRSR2.

Bioinformatics analysis and variant reporting: Sample demultiplexing and FASTQ file generation were performed in BaseSpace (Illumina). Alignment of reads to the reference genome (GRCh38) and variant calling was performed using the Dragen Enrichment pipeline (Illumina). VCF files were uploaded via the GenoVic GOS portal into Alissa Interpret for variant curation and reporting. The IGV browser was used to verify called variants and examine the nature and context of mapped reads. Variants were classified into five tiers of pathogenicity based primarily on the ACMG guidelines for germline mutations plus additional in‐house algorithms relevant to somatic variants. Benign and likely benign variants are not reported. Pathogenic, likely pathogenic, and variants of uncertain significance are reported. The variant allele frequency of reported variants is shown as a decimal value (i.e., 0.50 = 50%, 0.25 = 25%).

## Disclosures

Nothing to declare: Tomasz J. Block, Cat Shore‐Lorenti, Roger Zebaze, Peter G. Kerr, Anna Kalff, Frances Milat. Declared: Andrew Charles Perkins: Served on advisory boards for Novartis Oncology, Abbvie, CTI, and Sierra Oncology. Peter R. Ebeling: Has received research funding and honoraria from Amgen; and research funding from Sanofi, and Alexion.

## Funding Information

This manuscript received no specific grant from any funding agency in the public, commercial, or not‐for‐profit sectors.

## Author Contributions

**Tomasz J. Block:** Conceptualization; data curation; formal analysis; writing – original draft; writing – review and editing. **Cat Shore‐Lorenti:** Data curation; formal analysis; investigation; writing – review and editing. **Roger Zebaze:** Writing – review and editing. **Peter G. Kerr:** Investigation; writing – review and editing. **Anna Kalff:** Investigation; writing – review and editing. **Andrew Charles Perkins:** Investigation; writing – review and editing. **Peter R. Ebeling:** Conceptualization; data curation; formal analysis; investigation; methodology; supervision; writing – review and editing. **Frances Milat:** Conceptualization; data curation; formal analysis; investigation; methodology; supervision; writing – review and editing.

### Peer Review

The peer review history for this article is available at https://www.webofscience.com/api/gateway/wos/peer-review/10.1002/jbm4.10791.

## Data Availability

The data that support the findings of this study are available from the corresponding author upon reasonable request.
